# Spontaneous neural activity changes in minimal hepatic encephalopathy before and 1 month after liver transplantation

**DOI:** 10.3389/fnhum.2025.1682584

**Published:** 2025-11-05

**Authors:** Shiting Tang, Bin Qin, Yunli Zhang, Yunfei Wei, Jinyu Liang, Junli Liang, Zhijian Liang

**Affiliations:** ^1^Department of Neurology, The First Affiliated Hospital of Guangxi Medical University, Nanning, China; ^2^Department of Neurology, The Second Affiliated Hospital of Guangxi Medical University, Nanning, China; ^3^Department of Neurology, Liuzhou People's Hospital, Liuzhou, China; ^4^Department of Critical Care Medicine, People's Hospital of Guangxi Zhuang Autonomous Region, Nanning, China

**Keywords:** minimal hepatic encephalopathy, liver transplantation, dynamic low-frequency fluctuation, spontaneous neural activity, cognitive function

## Abstract

Minimal hepatic encephalopathy (MHE) is the initial stage of hepatic encephalopathy (HE), MHE patients have associated with widespread neuro-psychological impairment. Liver transplantation (LT) can restore metabolic abnormalities but the mechanisms are unclear. This study aimed to longitudinally evaluate brain function alteration in MHE patients one month after LT and their correlation with cognitive changes by using resting-state functional magnetic resonance imaging (rs-fMRI). Rs-fMRI data was collected from 32 healthy controls and 27 MHE before and 1 month after LT. Between-group comparisons of demographic data and neuropsychological scores were analyzed using SPSS 25.0. Functional imaging data were analyzed using RESTplus and SPM12 software based on MATLAB 2017b. Gender, age, and years of education were used as covariates to obtain low-frequency fluctuationd (ALFF) and dynamic low-frequency fluctuation (dALFF) dindices. Correlation analyses were performed to explore the relationship between the change of ALFF and dALFF with the change of clinical indexes pre- and post-LT. Compared to controls, ALFF values increased in the Left Cerebelum 8, right orbital part of the inferior frontal gyrus (ORBinf), right superior occipital gyrus (SOG) and decreased in right PreCG and left middle frontal gyrus (MFG) in patients post-LT; dALFF values increased in the right temporal pole and middle temporal gyrus (TPOmid), right ORBinf, left caudate nucleus (CAU), right SOG and decreased in left PreCG, left PCUN, left ANG, left SMA and left MFG in patients post-LT. Compared to pre-LT, ALFF values of post-LT patients increased in the right calcarine fissure and surrounding cortex (CAL), right MOG and decreased in right cerebelum 8, left PCUN; dALFF values of post-LT patients decreased in right thalamus (THA), left posterior cingulate gyrus (PCG) and left MFG. The changes of ALFF in the left PCUN, right CAL and right MOG were correlated with change of digit symbol test (DST) scores (*P* < 0.05). In summary, this study not only showcases the potential of ALFF/dALFF algorithms for assessing alterations in spontaneous neural activity in MHE, but also provides new insights into the altered brain functions in MHE patients 1 month after LT, which may facilitate the elucidation of elucidation of mechanisms underlying cognitive restoration post-LT in MHE patients.

## 1 Introduction

Minimal hepatic encephalopathy (MHE), recognized as the subclinical or incipient stage of hepatic encephalopathy, manifests solely as subtle neurocognitive impairments including attentional deficits, visuomotor dysfunction, impaired cognitive control, and disrupted working memory networks (Weissenborn, 2019). Affecting 20%-80% of cirrhotic patients, approximately 40% of untreated MHE cases progress to overt hepatic encephalopathy (OHE) within 6 months. The emergence of OHE portends substantially elevated mortality, exceeding 50% at 1 year and approaching 80% by 3 years post-onset, while severely compromising quality of life and imposing profound socioeconomic burdens on patients, families, and healthcare systems (Dhiman et al., 2010; Labenz et al., 2019; Weissenborn et al., 2004; Zhang et al., 2020).

Neurocognitive dysfunction in cirrhotic patients correlates with deteriorating daily functioning, impaired quality of life, and increased mortality (Córdoba, 2011). Liver transplantation (LT), the definitive therapeutic intervention for end-stage liver disease, enhances survival and life quality in this population (Cárdenas and Ginès, 2011). Concomitant with hepatic function recovery, significant cognitive improvement typically follows LT (Córdoba, 2011; Mechtcheriakov et al., 2004; Pujol et al., 1994). However, the extent of preoperative deficit reversibility remains contested. Mattarozzi et al. (2004) documented post-LT cognitive enhancement in MHE patients using neuropsychological batteries, noting improvements in visuospatial abilities, selective attention, and verbal memory (both short- and long-term) at 6 months, with further gains in selective attention and short-term verbal memory by 18 months. Conversely, Campagna et al. (2014) reported that although cognitive function improved post-LT in patients with prior OHE, it remained subnormal compared to non-OHE cirrhotic controls. Notably, Tryc et al. (2014) proposed that post-transplant cognitive impairment represents de novo deficits rather than residual dysfunction, exhibiting distinct features from HE-related impairment, with 70% of patients demonstrating decline in ≥1 domain on the Repeatable Battery for the Assessment of Neuropsychological Status (RBANS) at 1-year post-LT. Therefore, the underlying neuropathological mechanisms driving alterations in neurocognitive function after LT in MHE patients need further investigation.

Resting-state functional magnetic resonance imaging (rs-fMRI) has emerged as a pivotal tool for investigating the neural substrates of neuropsychiatric disorders (Guo et al., 2011; Liu et al., 2012; Palacios et al., 2013). The amplitude of low-frequency fluctuations (ALFF), first proposed by Zang et al. (2007), quantifies spontaneous neural activity by measuring energy within low-frequency oscillations. This metric has been extensively applied to detect regional neural alterations in MHE patients. Zhong et al. (2016) identified reduced ALFF in the precuneus and medial prefrontal cortex as potential diagnostic biomarkers for MHE, while Qi et al. (2012) reported decreased ALFF in default mode network (DMN) regions alongside elevated ALFF in the cerebellum and middle temporal gyrus in MHE patients. Critically, ALFF modifications correlate significantly with neurocognitive performance in MHE (Chen et al., 2016; Qi et al., 2012; Zhong et al., 2016). Notably, existing studies primarily characterize baseline cerebral activity in chronic disease states. Although Cheng et al. (2017) employed ALFF to evaluate spontaneous brain activity changes pre- vs. post-LT in cirrhotic patients, their cohort lacked MHE/OHE stratification. Furthermore, conventional rs-fMRI analyses, including ALFF, postulate static neural activity throughout scan durations. However, converging evidence demonstrates that the brain dynamically responds and adapts to intrinsic/extrinsic stimuli across multiple temporal scales (Xue et al., 2020). Dynamic low-frequency fluctuation (dALFF), an extension of traditional ALFF, addresses this limitation by segmenting the BOLD time-series into multiple windows with subsequent ALFF quantification per segment (Cui et al., 2020), thereby capturing whole-brain spatiotemporal dynamics. To date, integrated ALFF/dALFF analyses of early post-transplant neural reorganization remain scarce. Therefore, this study aims to characterize alterations in spontaneous brain activity in MHE patients 1 month after liver transplantation using concurrent ALFF and dALFF metrics.

## 2 Materials and methods

### 2.1 Participants

We recruited a consecutive series of cirrhotic patients who were scheduled for LT from March 2022 and January 2024. The inclusion criteria were as follows: (1) patients with a diagnosis of liver cirrhosis based on clinical history, biochemical findings, imaging findings, and/or histological examination, (2) diagnosis of minimal hepatic encephalopathy (MHE), (3) age between 18 and 60 years, (4) normal visual acuity, (5) right-handedness, and (6) without any contraindication to MRI scanning. Exclusion criteria for patient groups included: (1) history or current presentation of overt hepatic encephalopathy (OHE), (2) contraindications to MRI (e.g., implanted metallic devices, cardiac pacemakers) or claustrophobia, (3) presence of other intracranial organic pathologies identified on conventional MRI (e.g., cerebral infarction, brain tumors), (4) history of any drug or alcohol abuse, (5) concurrent severe cardiac, cerebral, pulmonary, or renal disease, (6) illiterate individuals or those unable to complete neuropsychological testing for other reasons. and (7) development of severe postoperative complications following liver transplantation, such as acute rejection, hepatic failure, significant biliary stricture, or other central nervous system complications (e.g., central pontine myelinolysis, cerebral infarction). We also recruited a healthy controls group, who were frequency matched in age, gender, and education using advertisements within our hospital. The control subjects had no history of neurologic, psychiatric, or traumatic diseases that could have affected brain function. All controls also had no liver or other systemic diseases.

This study was approved by the Ethics Committee of Guangxi Medical University, and we conducted all experiments in compliance with relevant guidelines and regulations. All participants provided written informed consent prior to the study.

### 2.2 Laboratory tests

Clinical laboratory parameters including blood ammonia, albumin, total bilirubin, and prothrombin time, were obtained for all patients during the week before or after MRI. Patients were instructed to refrain from smoking for 24 h prior to venipuncture. Blood samples were promptly refrigerated following collection. The Child–Pugh score was used to assess liver function, a higher score corresponds to a greater severity of hepatic impairment (Lv et al., 2021). Based on this aggregate score, hepatic functional reserve is stratified into Class A (5–6 points), Class B (7–9 points), and Class C (10–15 points). Laboratory tests were not performed for the HCs. In addition to a comprehensive self-reported medical history, all volunteers underwent a detailed structured interview conducted by a clinical researcher to explicitly exclude any history of liver disease, metabolic disorders, chronic illness, or regular medication use. Furthermore, we performed a physical examination focusing on signs of chronic liver disease (e.g., jaundice, spider angiomata). While we recognize that this does not completely eliminate the possibility of asymptomatic disease, this multi-layer screening approach is consistent with the methodology employed in several established MRI studies of healthy populationsthis multi-layer screening approach is consistent with the methodology employed in several established MRI studies of healthy populations (Cheng et al., 2021a).

### 2.3 Neuropsychological assessment

According to previous studies, the diagnosis of Minimal Hepatic Encephalopathy (MHE) was established according to criteria outlined in the report by the Working Party at the 11th World Congress of Gastroenterology (Ferenci et al., 2002) and the 2014 practice guidelines issued by the American Association for the Study of Liver Diseases and the European Association for the Study of the Liver (Vilstrup et al., 2014). The typical neuropsychological tests included the Number Connection Test-A (NCT-A) and the Digit Symbol Test (DST). Participants were diagnosed with MHE if their performance deviated by more than 2 standard deviations from the normative mean of the healthy control group on at least one of these tests (Cheng et al., 2021b; Groeneweg et al., 1998; Qi et al., 2012). All subjects underwent assessment using the NCT-A and DST scales under the supervision of a single, specifically trained physician within 1 h preceding their BOLD-fMRI scan.

### 2.4 MRI data acquisition

All imaging data were acquired using a Siemens 3.0T Tim Trio MRI scanner equipped with a 32-channel head coil. Subjects were positioned supine on the scanner bed, with head immobilization facilitated by sponge padding to minimize motion artifacts. Earplugs were provided to attenuate acoustic noise. The subjects were instructed to keep their eyes closed and stay awake during scanning.

The scanning protocol commenced with the acquisition of localizer images and conventional sequences, allowing subjects an adaptation period to acclimate to the scanner environment. The scanning protocol for each participant encompassed conventional T2-weighted imaging (T2WI), three-dimensional T1-weighted imaging (3D-T1WI), and rs-fMRI. The specific acquisition parameters for each sequence were as follows:

(1) T2WI: Employing a Fast Spin Echo (FSE) sequence; Repetition Time/Echo Time (TR/TE) = 4,500/107 ms; slice thickness = 5 mm; gap = 5 mm; Field of View (FOV) = 240 × 240 mm^2^; matrix size = 384 × 384; number of averages (NEX) = 1.

(2) 3D-T1WI: Utilizing a Magnetization Prepared Rapid Gradient Echo (MPRAGE) sequence for whole-brain volumetric acquisition, oriented sagittally; 176 slices; TR/TE = 2,000/1.9 ms; slice thickness = 1 mm; gap = 0 mm; Inversion Time (TI) = 950 ms; FOV = 256 × 256 mm^2^; matrix size = 256 × 256; flip angle = 8°; acquisition time = 3.27 min.

(3) rs-fMRI: Employing a Gradient-Recalled Echo Echo-Planar Imaging (GRE-EPI) sequence, oriented axially; 70 slices; TR/TE = 2,170/30 ms; slice thickness = 2 mm; gap = 0.3 mm; FOV = 192 × 192 mm^2^; matrix size = 96 × 96; flip angle = 90°; total dynamics = 186; acquisition time = 6.58 min.

### 2.5 Functional MRI imager preprocessing

Preprocessing was performed using the RESTplus toolkit and Statistical Parametric Mapping software (SPM12, Wellcome Trust Centre for Neuroimaging, London, UK) implemented in MATLAB R2017b (The MathWorks, Inc., Natick, MA, USA). To mitigate the influence of initial magnetic field inhomogeneity and subject acclimatization, the first 10 time points of each functional run were discarded. Subsequent analyses were performed on the remaining 176 time points. The motion-corrected functional images were then spatially normalized to the Montreal Neurological Institute (MNI) space by applying the parameters of structural image normalization and resampled to 3 mm isotropic voxels. The obtained data were subsequently smoothed with a 6 mm Gaussian kernel, band-pass filtered (0.01–0.08 Hz), and the linear trend removed.

Finally, fractional ALFF (fALFF) maps were generated by normalizing each voxel's ALFF value by the global mean ALFF value across the entire brain.

### 2.6 Computation of dynamic and static ALFF

The dynamic ALFF (dALFF) was estimated using a sliding window width of 50 TRs and a step size of 20 TRs [50 × (1–0.6)] (Cui et al., 2020; Liao et al., 2014; Zheng et al., 2021). This segmentation partitioned the entire time series per participant into 29 distinct temporal windows. Each window yielded a corresponding dALFF map. Temporal variability in intrinsic brain activity was subsequently assessed by computing the variance of the dynamic brain activity across these 29 windows. Finally, dALFF maps underwent normalization to z-scores for subsequent statistical analyses. Furthermore, to ascertain whether dALFF furnishes overlapping or complementary information relative to static measures, static ALFF values were also computed for each participant using the restplus toolbox. Following preprocessing, the time series for each voxel was subjected to band-pass filtering (0.01–0.08 Hz) to mitigate the influence of very low-frequency drift and high-frequency noise. The filtered time series was then transformed into the frequency domain via Fast Fourier Transform (FFT), yielding the frequency spectrum Y. The ALFF value was subsequently calculated according to the following formula:


$$\text{ALFF}=\sum_{\text{i = N1}}^{\text{N2}}\text{Yi}\text{/}{(}_{\text{N2}}-{\text{N1}}_{)}$$

where N1 and N2 denote the index positions in the discrete frequency spectrum corresponding to the lower and upper bounds, respectively, of the selected frequency band (0.01–0.08 Hz). ALFF values were computed for each group utilizing 176 temporal frequency bins within this band. The ALFF value for each voxel was normalized by dividing it by the global mean ALFF value across all voxels.

### 2.7 Statistical analysis

SPSS (Version 25.0) was used to perform statistical analyses of clinical and demographic characteristics. Chi-square (χ^2^) test were used to compare the sex differences. To evaluate disparities in age, years of education, and neuropsychological scores, independent samples *t-*tests and paired samples *t-*tests were employed for normally distributed data, while the Wilcoxon signed-rank test and the Mann–Whitney U test were used for non-normally distributed data. The level of statistical significance was set at p < 0.05. Statistical analysis of Resting-state functional MRI (rs-fMRI) data was using SPM (SPM12; http://www.fil.ion.ucl.ac.uk/spm). Two-sample *t-*tests was performed to compare the ALFF and dALFF values between the pre-operative Minimal Hepatic Encephalopathy (MHE) group vs. controls, and the post-operative MHE group vs. controls, with age, gender, and years of education incorporated as covariates. To further explore abnormality in ALFF/dALFF between the pre-LT and post-LT, paired *t-*tests was used. The multiple comparison correction was conducted using Gaussian Random Field (GRF) theory at thresholds of *P*~cluster~ < 0.05. After that, the ALFF/dALFF changes (displayed as ΔALFF/dALFF) in brain regions that were significantly different between pre- and post-LT were extracted from each patient. The correlation between ΔALFF/dALFF and the changes in corresponding clinical indexes (e.g., ΔNCT-A, ΔDST, Δprothrombin time, Δalbumin, Δtotal bilirubin and Δammonia) were calculated using the Pearson correlation analysis. The threshold of significance was set at *p* < 0.05.

## 3 Results

### 3.1 Demographic and clinical data

There are 31 MHE patients received LT operation, but during the first month (28 ± 5 days) follow-up examination, one patients dropped out of this study because of death, three complications and refusal. Thus, 27 patients (mean age: 46.9 ± 9.1 years, female/male = 6/21) completed all preand post-operative MRI procedures and cognitive functiontests. Etiological distribution of cirrhosis included hepatitis B (*n* = 15), hepatitis C (*n* = 4), cholestatic liver disease (*n* = 5), and cryptogenic cirrhosis (*n* = 3). Child-Pugh classification revealed Grade A in 2 patients, Grade B in 5, and Grade C in 20. Demographics and clinical data for all subjects are summarized in Table 1. There were no meaning differences in sex, age, or education level between MHE and HC groups (*p* values > 0.05). For MHE groups, liver function improved significantly (prothrombin time, albumin, total bilirubin, blood ammonia, *p* < 0.05) or showed a tendency toward restoration 1 month after LT.

**Table 1:** Demographic, neuropsychological, and clinical date.

**Parameter**	**HC (*n =* 32)**	**Pre-MHE (*n =* 27)**	**Post-MHE (*n =* 27)**	***p***		
				**Pre-MHE/HC**	**Post-MHE/HC**	**Post-MHE/ Pre-MHE**
Sex (male/famale)	25/7	21/6	21/6	0.974^*^	0.974^*^	-
Age (year)	45.8 ± 8.5	46.9 ± 9.1	46.9 ± 9.1	0.661^#^	0.661^#^	-
Education (year)	9.0 (8.0–11.0) (12.3)	9.0 (9.0–12.0)	9.0 (9.0–12.0)	0.246^b^	0.246^b^	-
NCT-A (second)	43.7 ± 8.3	76.6 ± 10.6	51.1 ± 5.0	0.000^#^	0.000^#^	0.000^%^
DST (score)	47.7 ± 7.9	33.4 ± 8.3	43.3 ± 5.5	0.000^#^	0.018^#^	0.000^%^
**Laboratory examination**						
Prothrombin time (second)	-	13.2 (12.3–18.7)	12.4 (11.3–14.2)	-	-	0.011^a^
Albumin (mg/dL)	-	33.3 ± 7.2	38.9 ± 4.2	-	-	0.002^%^
Total bilirubin (mg/dL)	-	33.8 (20.8–134.0)	13.1 (10.3–22.3)	-	-	0.001^a^
Blood ammonia (μmol/L)	-	145.6 ± 32.3	46.2 ± 10.4	-	-	0.000^%^
Child-Pugh A/B/C	-	2/5/20	-	-	-	-

Data are presented as mean ± standard deviation or Median (IQR).

^*^The *p* value was obtained by the chi-square test.

^#^The *p* value was obtained by the One-sample *T*-test.

^%^The *p* value was obtained by the paired sample *T*-test.

^a^The *p* value was obtained by the Wilcoxon signed-rank test.

^b^The *p* value was obtained by the Mann-Whitney *U* test.

As expected, pre-LT group underperformed compared with HC took longer to complete NCT-A tasks (*p* values < 0.05) and had lower scores in DST tasks (*p* values < 0.05). One month after LT, compared with HCs, pre-LT group showed comparable performance in both DST (*p* values < 0.05) and NCT-A (*p* values < 0.05) tasks. The increased performance in DST scores and decreased performance in NCT-A scores suggests an enhancement of cognitive capability. Compared with the HCs, the DST and NCT-A scores in the post-LT group remained worse, indicating incomplete restoration of cognitive function.

### 3.2 Group different in ALFF and dALFF

**Figure 1:**
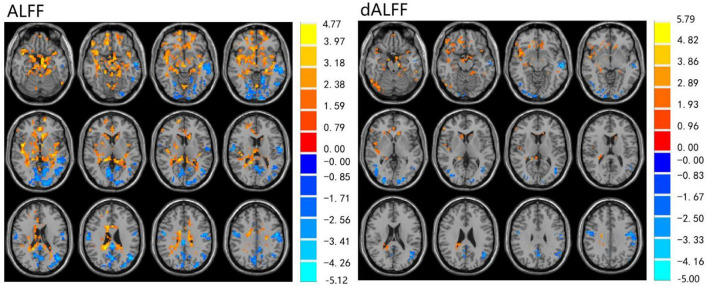
Differences in ALFF and dALFF between the pre-LT and HCs, voxel level *p* < 0.05, cluster level *p* < 0.05 (GRF correction).

**Figure 2:**
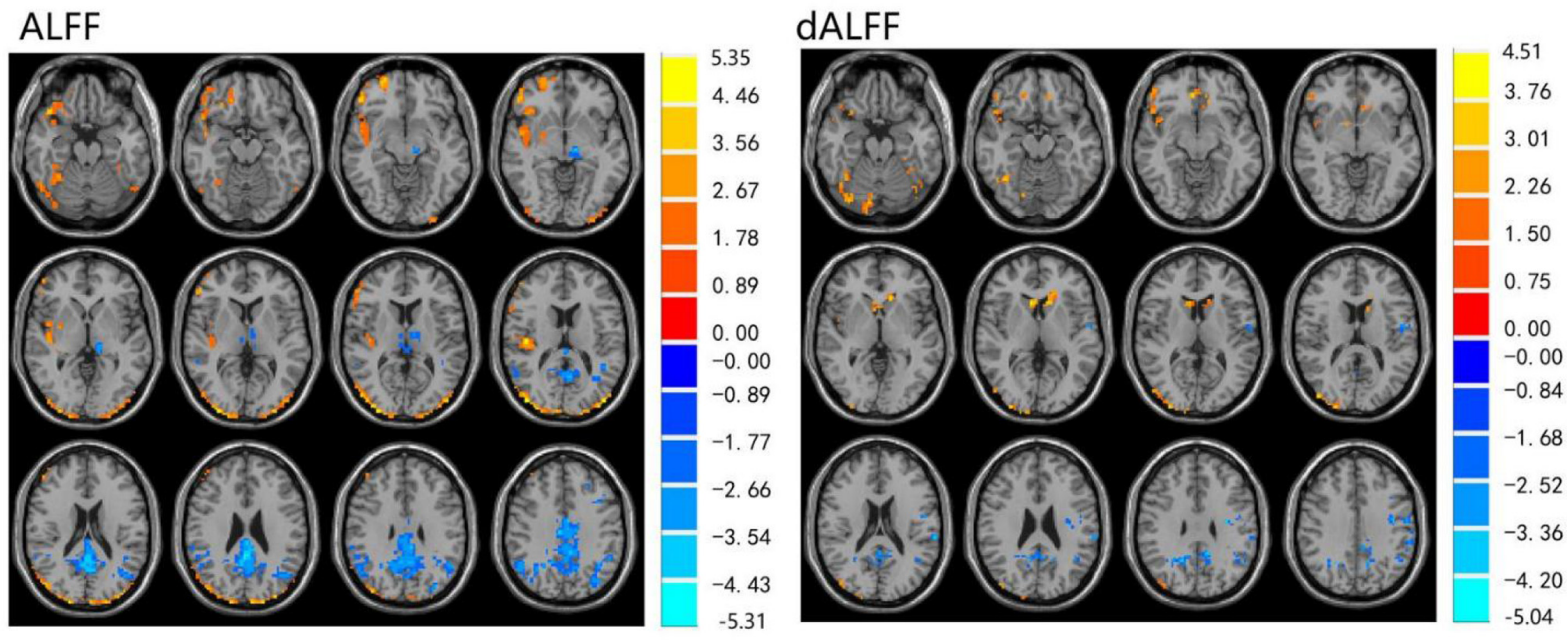
Differences in ALFF and dALFF between the post-LT and HCs, voxel level *p* < 0.05, cluster level *p* < 0.05 (GRF correction).

**Figure 3:**
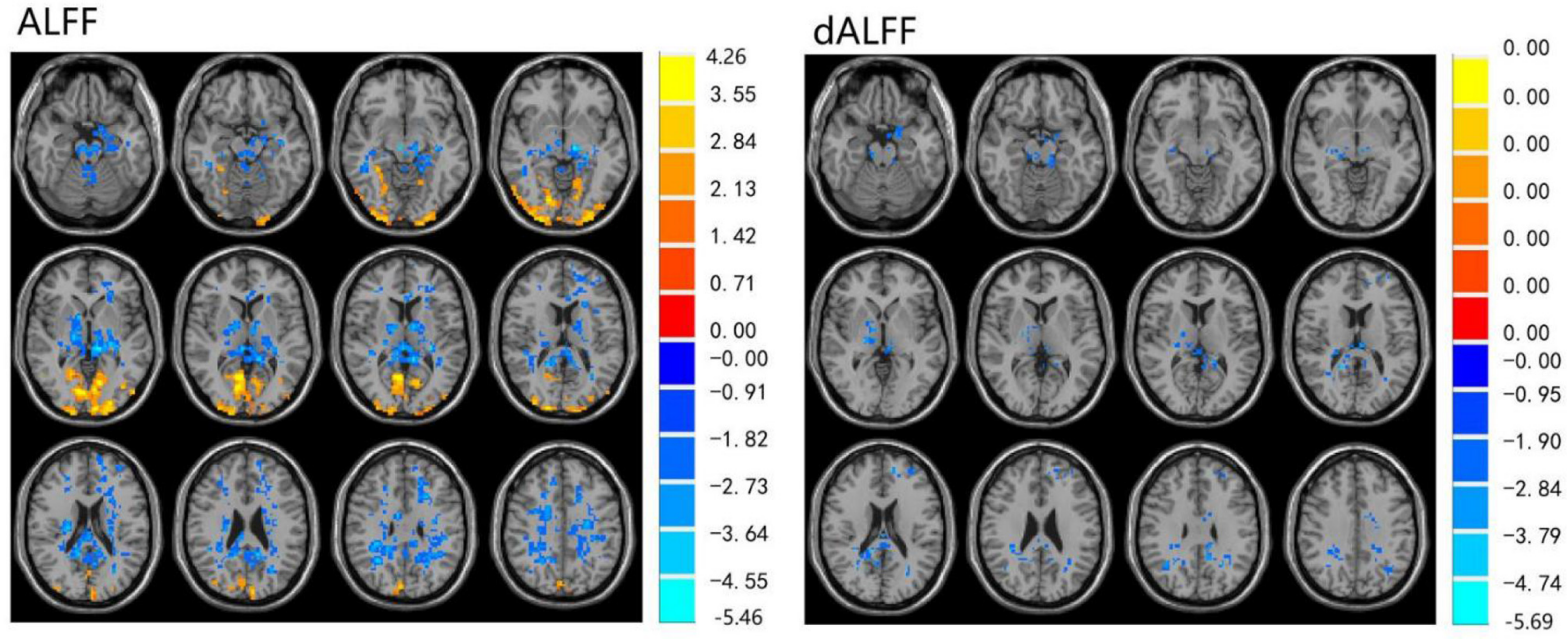
Differences in ALFF and dALFF between the post-LT and pre-LT, voxel level *p* < 0.05, cluster level *p* < 0.05 (GRF correction).

The ALFF differences between pre- and post-LT group and the HCs and between the post- and pre-LT groups are displayed in Tables 2–4 and Figures 1–3. Compared with the HCs, the pre-LT group showed significantly decreases ALFF in the left middle temporal gyrus, left precentral gyrus, and right precentral gyrus (Table 2 and Figure 1). The post-LT group displayed increased ALFF in the left cerebellar lobule VIII, right pars orbitalis of the inferior frontal gyrus, and right superior occipital gyrus and reduced ALFF in the right precentral gyrus and left middle frontal gyrus (Table 3 and Figure 2). Interestingly, to further delineate the impact of LT on brain activity, paired *t-*tests were performed between pre- and post-LT groups. After LT, the post-LT patients demonstrated significant ALFF increases in the right pericalcarine cortex and right middle occipital gyrus and ALFF decreases in the right cerebellar lobule VIII and left precuneus (Table 4 and Figure 3).

**Table 2:** Brain regions showed differences in ALFF/dALFF of in the comparisons between pre-LT and HCs.

**Imaging metrics**	**Brain regions (ALL)**	**MNI coordinate (mm)**			**Voxel number**	**Peak *t* value**
		**X**	**Y**	**Z**		
ALFF	Middle temporal gyrus (left)	3	6	60	90	−5.1159
	Precentral gyrus (left)	−51	−9	33	42	−4.5314
	Precentral gyrus (right)	60	−9	15	127	−3.6833
dALFF	Cerebellar lobule VIII (left)	−6	−24	−54	40	4.5017
	Inferior temporal gyrus (left)	−45	−66	−30	25	4.1065
	TemporalPole: middle temporal gyrus (right)	48	15	−30	60	5.7897
	Cerebellar lobule VIII (right)	12	−66	−39	16	3.5631
	Parahippocampal gyrus (left)	6	−18	−24	38	4.3087
	Middle temporal gyrus (left)	−60	−21	−9	71	−4.9967
	Inferior temporal gyrus (right)	57	−60	−21	17	4.373
	Inferior occipital gyrus (right)	27	−93	0	18	−3.3721
	Middle occipital gyrus (left)	−21	−90	0	32	−3.3492
	Middle temporal gyrus (right)	42	−66	3	26	−3.694
	Angular gyrus (left)	−45	−60	3	35	−3.2351
	Precuneus (left)	−6	−45	48	29	−3.3033
	Precentral gyrus (right)	48	−9	39	40	−3.4284
	Precentral gyrus (left)	−57	−12	33	36	−4.841
	Supplementary motor area (left)	3	9	66	29	−3.6593
	Supplementary motor area (right)	3	−30	60	23	−3.6703

Voxel level *p* < 0.05, cluster level *p* < 0.05 (GRF correction).

**Table 3:** Brain regions showed differences in ALFF/dALFF of in the comparisons between post-LT and HCs.

**Imaging metrics**	**Brain regions (ALL)**	**MNI coordinate (mm)**			**Voxel number**	**Peak *t* value**
		**X**	**Y**	**Z**		
ALFF	Cerebellar lobule VIII (left)	−36	−36	−30	43	3.6365
	Pars orbitalis: inferior frontal gyrus (right)	45	−18	18	118	4.9811
	Superior occipitals gyrus (right)	24	−99	18	31	5.3465
	Precentral gyrus (ringht)	−33	−78	42	290	−5.3101
	Middle frontal gyrus (left)	−42	18	45	145	−4.8178
dALFF	TemporalPole: middle temporal gyrus (right)	48	15	−30	36	4.5134
	Pars orbitalis: inferior frontal gyrus (right)	51	36	−12	45	3.1821
	Caudate nucleus (left)	9	21	6	23	3.9663
	Superior occipital gyrus (right)	36	−93	18	39	4.1433
	Precentral gyrus (left)	−33	−12	30	11	−3.6477
	Precuneus (left)	−12	−48	33	62	−4.2179
	Angular gyrus (left)	−42	−63	42	46	−3.9301
	Supplementary motor area (left)	−3	−3	57	47	−3.7519
	Middle frontal gyrus (left)	−42	15	42	88	−4.3274

Voxel level *p* < 0.05, cluster level *p* < 0.05 (GRF correction).

**Table 4:** Brain regions showed differences in ALFF/dALFF of in the comparisons between post-LT and pre-LT.

**Imaging metrics**	**Brain regions (ALL)**	**MNI coordinate (mm)**			**Voxel number**	**Peak *t* value**
		**X**	**Y**	**Z**		
ALFF	Cerebellar lobule VIII (right)	9	−63	−45	55	−3.8761
	Precuneus (left)	−12	−30	0	60	−5.4636
	Calcarine sulcus pericalcarine cortex (right)	12	−63	6	150	4.2609
	Middle occipital gyrus (right)	24	−102	−3	57	4.0317
dALFF	Thalamus (right)	−18	3	−24	32	−4.4422
	Posterior cingulate cortex (left)	9	−36	12	19	−5.6886
	Middle frontal gyrus (left)	−24	42	27	43	−3.72

Voxel level *p* < 0.05, cluster level *p* < 0.05 (GRF correction).

For the dALFF, two-group *t-*test of pre-LT group showed significantly increased dALFF in the left cerebellar lobule VIII, left inferior temporal gyrus, right temporal pole extending to the middle temporal gyrus, right cerebellar lobule VIII, left parahippocampal gyrus, right inferior temporal gyrus and decreased dALFF in the left middle temporal gyrus, right inferior occipital gyrus, left middle occipital gyrus, right middle temporal gyrus, left angular gyrus, left precuneus, right precentral gyrus, left precentral gyrus, left supplementary motor area, and right supplementary motor area compared to control group (Table 2 and Figure 1). Compared with the HCs, the post-LT group still showed some abnormal areas with increased dALFF in the right temporal pole: middle temporal gyrus, right pars orbitalis of the inferior frontal gyrus, left caudate nucleus, right superior occipital gyrus, and decreased dALFF in the left precentral gyrus, left precuneus, left angular gyrus, left supplementary motor area, and left middle frontal gyrus (Table 3 and Figure 2). Paired *t-*tests demonstrated significant dALFF reductions in the right thalamus, left posterior cingulate cortex, and left middle frontal gyrus compared to preoperative states (Table 4 and Figure 3).

### 3.3 Correlation analyses

**Figure 4:**
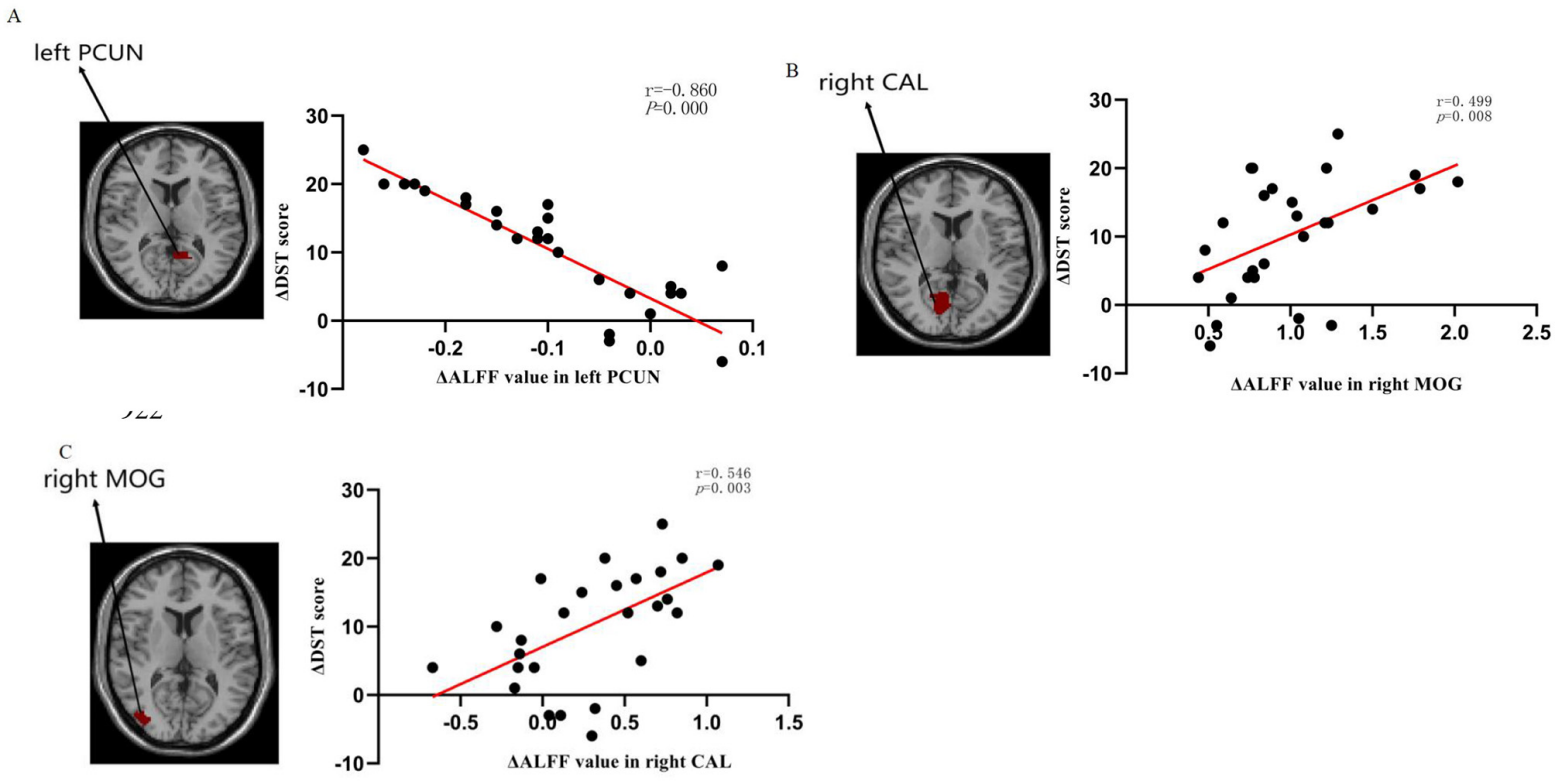
Relationship of changes in amplitude of low-frequency fluctuation (ALFF) values with changes in DST performance: **(A)** The ΔALFF in the left PCUN was negatively correlated with DST performance (*r* = −0.860, *P* = 0.000), **(B)** The ΔALFF in the right CAL was positively correlated with DST performance (*r* = 0.546, *P* = 0.003), **(C)** The ΔALFF in the right MOG was positively correlated with DST performance (*r* = 0.499, *P* = 0.008). ALFF low-frequency fluctuation, DST digit-symbol test, PCUN precuneus, CAL calcarine fissure, and surrounding cortex, MOG middle occipital gyrus.

We examined the correlation between ΔALFF/dALFF and ΔNCTA, ΔDST scores, Δprothrombin time, Δalbumin, Δtotal bilirubin and Δammonia (Table 5 and Figure 4). Bonferroni correction was used for the multiple comparisons when performing correlation analysis (7 brain regions showed significantly altered ΔALFF/dALFF pre- and post-LT). ΔALFF in the in the right pericalcarine cortex (^*^r^*^ = 0.546, *P* = 0.003) and the right middle occipital gyrus (^*^r^*^ = 0.499, *P* = 0.008) were positively correlated with ΔDST scores. While ΔALFF in the left precuneus (^*^r^*^ = −0.860, *P* < 0.001) was negative correlated with ΔDST scores. No significant correlations were observed between other regions and neuropsychological or biochemical indices.

**Table 5:** Correlations between the changes of ALFF/dALFF and changes of NCT-A, DST scores, prothrombin time, albuminand, total bilirubin, and ammonia 1-month post- and pre-LT.

**Imaging metrics**	**ROIs**		**CCs between ΔALFF/ ΔdALFF and ΔNCT –A (seconds)**	**CCs between ΔALFF/ ΔdALFFF and ΔDST**	**CCs between ΔALFF/ ΔdALFF and Δprothrombin time**	**CCs between ΔALFF/ ΔdALFF and Δalbuminand**	**CCs between ΔALFF/ ΔdALFF and Δtotal bilirubin**	**CCs between ΔALFF/ ΔdALFF and Δammonia**
ALFF	Cerebellar lobule VIII (right)	−0.09 ± 0.02	*r =* 0.054 *P =* 0.790	*r = –*0.188 *P =* 0.347	*r = –*0.097 *P =* 0.631	*r =* 0.114 *P =* 0.571	*r =* 0.146 *P =* 0.469	*r = –*0.080 *P =* 0.693
	Precuneus (left)	−0.10 ± 0.02	*r =* 0.067 *P =* 0.741	*r = –*0.860 *P =* 0.000^*^	*r = –*0.067 *P =* 0.739	*r = –*0.96 *P =* 0.633	*r = –*0.055 *P =* 0.783	*r =* 0.127 *P =* 0.527
	Calcarine sulcus pericalcarine cortex (right)	0.30 ± 0.08	*r = –*0.108 *P =* 0.592	*r =* 0.546 *P =* 0.003^*^	*r = –*0.221 *P =* 0.269	*r = –*0.062 *P =* 0.759	*r =* 0.135 *P =* 0.500	*r = –*0.090 *P =* 0.657
	Middle occipital gyrus (right)	1.00 ± 0.08	*r = –*0.136 *P =* 0.498	*r =* 0.499 *P =* 0.008^*^	*r = –*0.119 *P =* 0.554	*r =* 0.134 *P =* 0.506	*r = –*0.022 *P =* 0.913	*r =* 0.189 *P =* 0.345
dALFF	Thalamus (right)	−0.04 ± 0.01	*r = –*0.245 *P =* 0.219	*r =* 0.085 *P =* 0.673	*r = –*0.190 *P =* 0.341	*r =* 0.195 *P =* 0.330	*r = –*0.162 *P =* 0.419	*r =* 0.03 *P =* 0.988
	Posterior cingulate cortex (left)	−0.02 ± 0.01	*r = –*0.349 *P =* 0.074	*r = –*0.246 *P =* 0.216	*r = –*0.066 *P =* 0.743	*r =* 0.369 *P =* 0.058	*r = –*0.091 *P =* 0.652	*r = –*0.334 *P =* 0.89
	Middle frontal gyrus (left)	−0.03 ± 0.01	*r =* 0.014 *P =* 0.946	*r =* 0.147 *P =* 0.465	*r = –*0.172 *P =* 0.392	*r =* 0.300 *P =* 0.129	*r = –*0.280 *P =* 0.157	*r =* 0.256 *P =* 0.197

ALFF, low-frequency fluctuationd; dALFF, dynamic low-frequency fluctuationd; LT, liver transplantation; ROI, region of interest; CC, correlation coefficient; NCT-A, number connection test-A; DST, digit-symbol test.

Δ Represents for post and pre LT difference of values.

^*^Represents for *P* < 0.05 uncorrected.

## 4 Discussion

In this study, we applied ALFF and dALFF together to investigate the change of brain function in MHE patients after LT. Specifically, we found that ALFF and dALFF had a similarity and complementarity effect on spontaneous brain activity. Compared wish HCs, the pre-MHE group exhibited decreased ALFF in the left middle temporal gyrus and bilateral precentral gyri. Excepted the above areas, the pre-MHE group also showed decreased dALFF in the right inferior occipital gyrus, left middle occipital gyrus, right middle temporal gyrus, left angular gyrus, left precuneus, and bilateral supplementary motor areas. Furthermore, there were increased dALFF in bilateral cerebellar lobule VIII, bilateral inferior temporal gyri, right temporal pole extending to middle temporal gyrus, and left parahippocampal gyrus. These findings suggest that spontaneous brain activity of most brain regions with abnormal ALFF/dALFF value before LT were substantially improved and nearly normalized at 1 month after LT, but spontaneous brain activity of some brain regions with decreased ALFF/dALFF in pre-LT was persistently existence, such as left cerebellar lobule VIII, bilateral precentral gyri, left precuneus, left angular gyrus, right temporal pole/middle temporal gyrus junction, and left supplementary motor area. Furthermore, there were new-onset ALFF/dALFF increased in right pars orbitalis of inferior frontal gyrus and right superior occipital gyrus; ALFF increased in right middle occipital gyrus and right pericalcarine cortex; ALFF/dALFF decreased in left middle frontal gyrus; dALFF decreased in left cingulate gyrus and right thalamus. To sum up, ALFF/dALFF had practical value in detecting the brain function changes after LT, and the partial renormalization of spontaneous brain activity and complete cognitive function recovery may need more time.

Although the exact biologic mechanisms of ALFF are still unclear, many studies have suggested that altered ALFF is associated with abnormal regional neuronal activity (Qi et al., 2012; Zhang et al., 2010). Compared with other methods, the strength of ALFF lies in its directly reflecting the amplitude of spontaneous brain activity. ALFF has been widely utilized in studies of neuropsychological diseases including Hepatic Encephalopathy (HE) (Chen et al., 2016; Lv et al., 2013; Qi et al., 2012). In this study, we found that abnormal ALFF in MHE patients before and after LT suggests impaired neural functioning in specific brain regions, although these findings are not entirely consistent with previous research. This discrepancy may be attributable to differences in the study populations. Although Cheng et al. (2017) and Zhang et al. (2017b) similarly investigated the short-term effects of LT on ALFF in cirrhotic patients, Cheng et al. focused on end-stage cirrhosis, Zhang et al. examined cirrhosis with or without OHE, whereas the present study specifically explored MHE patients, additionally utilized the dALFF analytical approach. Compared to ALFF, dALFF can reveal the adaptability and flexibility of spontaneous brain activity by capturing changes in resting-state energy consumption (Jiang et al., 2020), thereby further elucidating the time-varying nature of regional brain activity.

Concurrently, our study revealed that aberrant ALFF and dALFF observed in most brain regions pre-LT demonstrated reversal at 1-month post-LT. These regions encompassed the bilateral middle temporal gyri (MTG), inferior temporal gyri (ITG), and inferior occipital gyri (IOG), alongside the left parahippocampal gyrus. Notably, the IOG plays a pivotal role in visual information processing and visuospatial integration (Lee et al., 2000; Wandell et al., 2007; Yue et al., 2023). This finding holds clinical relevance given that visual dysfunction constitutes a hallmark feature in MHE patients (Amodio et al., 2004; Arias et al., 2015). The occipital lobe, particularly the IOG, serves as a critical hub within the visual network responsible for information integration and processing (Wang et al., 2019). This aligns with multiple resting-state fMRI studies reporting varying degrees of impairment within the visual cortex of MHE (Bajaj et al., 2017; Wang et al., 2019; Zhang et al., 2017a). Furthermore, Ji et al. (2023) reported a positive correlation between elevated dALFF values in the left inferior occipital gyrus (IOG) and blood ammonia levels. Ammonia represents one of the principal neurotoxins in the brain, and its detoxification increases glutamine levels within astrocytes, leading to cellular edema and ultimately contributing to functional impairment (Córdoba and Mínguez, 2008). Consequently, we speculate that the observed visual-related deficits in MHE patients—such as impairments in visual memory, visuomotor function, and visuospatial reasoning—may arise from ammonia-induced cellular damage, which subsequently disrupts neural activity within the occipital lobe. This study demonstrated that normalization of ALFF and dALFF in visual-associated regions following liver transplantation corresponded with substantial improvements in visual information processing capacity among MHE patients. This finding indicates that LT may enhance early postoperative visual function in this population. Furthermore, these spontaneous brain activity alterations were accompanied by significant cognitive improvement. Post-LT assessments revealed robust enhancements in both neuropsychological tests. Notably, we identified significant correlations between ΔALFF values and changes in Digit Symbol Test (DST) scores: Positive correlations in the right paracentral lobule (*r* = 0.546, *p* = 0.003) and right middle occipital gyrus (*r* = 0.499, *p* = 0.008). Negative correlation in the left precuneus (*r* = –0.860, *p* < 0.001). The current study found a significant correlation between ΔALFF values and performance on the Digit Symbol Test (DST), but not with the Number Connection Test-A (NCT-A) or conventional liver function indicators. This discrepancy may be attributed to several factors. First, the DST is a highly sensitive tool that assesses processing speed, visual-motor coordination, and sustained attention, domains often affected in the early stages of MHE. In contrast, the NCT-A primarily measures psychomotor speed and is considered by some studies to be less sensitive to subtle cognitive changes compared to the DST (Randolph et al., 2009; Weissenborn et al., 2001). Second, the lack of significant findings with NCT-A and liver indicators might be due to the limited statistical power resulting from our moderate sample size. A larger cohort might be necessary to detect weaker, yet potentially clinically relevant, associations. Future studies with a larger sample size and a broader battery of neuropsychological tests are warranted to confirm and extend our findings. These region-specific ALFF changes may serve as quantifiable neuroimaging markers reflecting the degree of cognitive recovery in MHE throughout the LT process.

Furthermore, this investigation revealed persistent aberrant neural activity in specific regions post-LT, including the left cerebellar Crus I/II (lobule VIII), bilateral precentral gyri (PreCG), left precuneus, left angular gyrus (AG), right temporal pole extending to the middle temporal gyrus (MTG), and left supplementary motor area (SMA). The functional significance of these regions is multi-faceted: Cerebellar Crus I/II: Beyond regulating balance, muscle tone, and motor coordination, this region modulates cognitive and motor functions via cerebello-cortical circuits (Lewis et al., 2004; Saalmann and Kastner, 2015; Schmahmann and Caplan, 2006; Stoodley and Schmahmann, 2010; Xiao and Scheiffele, 2018). Frontal Lobe Components (PreCG and SMA): As integral elements of the frontal lobe, damage to these areas may induce memory deficits, language impairments, or other cognitive dysfunctions (Yuan et al., 2016). The SMA specifically governs internally generated movement rather than externally cued actions (Shima and Tanji, 1998), suggesting that SMA dysfunction may underlie diminished volitional control in MHE patients. Default Mode Network (DMN) Hubs:Precuneus: Facilitates high-level integrative processes including episodic memory retrieval, visuospatial attention, and self-referential processing (Cavanna and Trimble, 2006) Middle Temporal Gyrus (MTG): Supports episodic memory consolidation (Dickerson et al., 2004) Angular Gyrus (AG): Serves as a critical nexus for attentional allocation, visual integration, and reading comprehension (Bocca et al., 2015). Post-liver transplantation, sustained reductions in ALFF/dALFF were observed in motor-associated regions (bilateral precentral gyri, left supplementary motor area) and Default Mode Network hubs (left precuneus, left angular gyrus). Conversely, persistent elevations emerged in the left cerebellar Crus I/II and right temporopolar-middle temporal continuum—interpreted as compensatory neuroplastic reorganization. These findings suggest that while LT significantly reduces neurotoxins and enables partial functional recovery, irreversible cellular damage from pre-existing metabolic dysfunction persists, thereby perpetuating residual cognitive and psychomotor deficits. This pathophysiological continuum aligns with established evidence of permanent neural injury in chronic hepatic encephalopathy (Lin et al., 2014; Mattarozzi et al., 2012; Mechtcheriakov et al., 2004).

Notably, compared to the pre-LT group, the post-LT group exhibited newly emerged reductions in dALFF within the left middle frontal gyrus (MFG). Similarly, relative to healthy controls, diminished ALFF and dALFF values were observed in the same region post-LT. This aligns with electrophysiological evidence from Blauenfeldt et al. (2010) suggesting cortical reorganization in MHE. We thus postulate that these emergent alterations in MFG may reflect post-transplant neurofunctional reorganization in MHE patients. Although our study did not find a direct correlation between left middle frontal gyrus activation and a specific cognitive test at this time point, the well-documented role of this region in executive control (Kim et al., 2012; Niendam et al., 2012) strongly suggests that its functional state is highly relevant to postoperative cognitive recovery. The persistence of hypoactivation may indicate a vulnerability in executive functions that could be explored in future studies with more targeted behavioral assessments. While the exact etiology remains undetermined, potential contributors include: postoperative homeostatic dysregulation, systemic infections, immunosuppressive pharmacotherapy, and other unidentified perioperative stressors, the mechanistic validation requires targeted exploratory investigations in future.

This study has several limitations. First, the absence of cardiorespiratory monitoring may permit physiological noise contamination in low-frequency fMRI signals, potentially introducing confounds in statistical analyses. Second, etiological heterogeneity within the cirrhotic cohort could engender classification bias, given that distinct disease etiologies may exert differential effects on neuropathological trajectories. Third, restriction to a single early postoperative timepoint precludes characterization of longitudinal neuromodulatory dynamics; serial assessments across extended recovery phases would provide greater insight into cognitive and neurofunctional evolution post-LT, that is a critical direction for future investigation.

## 5 Conclusion

In summary, we found that decreased intrinsic brain activity in the vision-related can be reversed 1 month after LT, indicating that LT can improve brain function. However, persistent ALFF/dALFF aberrations persist in motor-associated regions (bilateral precentral gyri, left supplementary motor area) and Default Mode Network components (left precuneus, left angular gyrus), suggesting continued abnormalities in these functional domains. Notably, emergent hypoactivation in the left middle frontal gyrus signifies potential de novo cognitive reorganization post-LT. Thus, this study not only showcases the potential of ALFF/dALFF algorithms for assessing alterations in spontaneous neural activity in MHE, but also provides new insights into the altered brain functions in MHE patients 1 month after LT, which may facilitate the elucidation of elucidation of mechanisms underlying cognitive restoration post-LT in MHE patients.

## Data Availability

The original contributions presented in the study are included in the article/supplementary material, further inquiries can be directed to the corresponding authors.
